# Uridine Cytidine Kinase Like-1 Enhances Tumor Cell Proliferation and Mediates Protection from Natural Killer-Mediated Killing

**DOI:** 10.23937/2378-3672/1410018

**Published:** 2016-05-24

**Authors:** Gail Gullickson, Elise C Ambrose, Richard G Hoover, Jacki Kornbluth

**Affiliations:** 1 Department of Pathology, Saint Louis University School of Medicine, USA; 2 VA, St. Louis Health Care System, St. Louis, USA

**Keywords:** Uridine kinase, Natural killer cells, Apoptosis, NF-Kb, Cell mediated cytotoxicity, Ubiquitin ligase

## Abstract

Uridine cytidine kinase like-1 (UCKL-1) is a largely uncharacterized protein over-expressed in many tumor cells, especially in highly malignant, aggressive tumors. Sequence analysis indicates that UCKL-1 has homology to uridine kinases, enzymes that play a role in DNA and RNA synthesis and that are often up-regulated in tumor cells. Previous studies have shown that UCKL-1 is a substrate for natural killer lytic-associated molecule (NKLAM), an E3 ubiquitin ligase found in NK cell cytolytic granules. Ubiquitination of UCKL-1 by NKLAM leads to its degradation. Increased expression of NKLAM enhances NK-mediated tumoricidal activity. The fact that UCKL-1 is a substrate for NKLAM suggests that UCKL-1 may provide resistance to NK killing in tumor cells. Here we show that UCKL-1 over-expression protects tumor cells from NK killing and enhances tumor survival *in vivo*. UCKL-1 also has a much broader role, protecting tumor cells from spontaneous and drug-induced apoptosis and increasing tumor cell proliferation. Nuclear factor-kappa B (NF-κB) activity is higher in tumor cells transfected with UCKL-1 compared to control transfected cells, suggesting at least one possible mechanism by which UCKL-1 influences tumor growth and survival.

## Introduction

Uridine cytidine kinase like-1 (UCKL-1) is a novel protein that has been largely unstudied. It was first identified as a protein that binds to Epstein-Barr virus encoded nuclear protein EBNA-3 [1]. UCKL-1 has homology to uridine kinases, which catalyze the phosphorylation of uridine to UMP, a rate limiting step in DNA and RNA synthesis [2]. Uridine kinases are important in cellular proliferation and survival and are often up-regulated in tumor cells [3].

UCKL-1 interacts with natural killer lytic-associated molecule (NKLAM), a protein associated with natural killer (NK) cell-mediated anti-tumor activity. NK cells play an important role in the tumor-host interaction and are a crucial element of the innate immune response and early recognition of tumors [4]. NK cells are lymphocytes that act as an important link between innate and adaptive immunity by contributing to the initiation of tumor specific immune responses [5]. NK cells kill tumor cells via the recognition of NK receptor ligands on tumor cells [5–9]. Once the ligand is recognized, NK granules migrate to the site of interaction between the NK cell and the tumor cell. NKLAM is rapidly synthesized by NK cells and is targeted to cytolytic granule membranes [10]. Granule components, including perforin and granzymes, are released into the intracellular junction, resulting in target cell apoptosis [5,11]. NKLAM is also released upon NK degranulation and is critical for maximal NK killing activity [10]. Conversely, diminished killing activity is seen in NKLAM-deficient NK cells and in NKLAM-deficient knockout (KO) mice [12].

NKLAM is a member of the RING-in between RING-RING (RBR) family of E3 ubiquitin ligases. These proteins regulate the ubiquitination of proteins, catalyzing the transfer of activated ubiquitin from an E2 ubiquitin-conjugating enzyme to its substrate. UCKL-1 is a substrate for NKLAM. UCKL-1 translocates from the nucleus to the cytoplasm in the presence of NKLAM, where it is ubiquitinated and degraded [13]. Enhanced expression of NKLAM, with concomitant reduction in UCKL-1 within the tumor cell, results in more tumor cell lysis.

Microarray analyses indicate that UCKL-1 levels are higher in many tumor cells compared to normal cells. UCKL-1 was identified as a top gene differentially expressed in benign versus malignant prostate cancer, with highest expression associated with the malignant phenotype [14]. Proteomic analysis identified UCKL-1 as a potential breast cancer biomarker, with its expression high in transformed cells and further increased in later stages of the disease [14,15]. Over-expression of UCKL-1 was also found to be a component of a genetic profile predictive of the development of therapy-induced acute myeloid leukemia among pediatric acute lymphoblastic leukemia patients [16]. Our previous studies demonstrated that down-regulation of UCKL-1 in K562 human tumor cells by RNA interference causes a decrease in proliferation, an increase in spontaneous apoptosis and in the susceptibility to lysis by NK cells. UCKL-1 down-regulation also results in enhanced susceptibility to death induced by the chemotherapeutic drugs staurosporine and etoposide [17].

Based upon our observations and those of others, the present study was initiated to further elucidate the role of UCKL-1 in tumor survival *in vitro* and *in vivo*. Studies to determine a potential mechanism by which UCKL-1 influences cell growth and apoptosis focused on nuclear factor-kappaB (NF-κB). NF-κB regulates the expression of genes that influence tumor growth, metastasis, apoptotic activity, and innate and adaptive immune responses [18–20]. The p65 subunit of NF-κB contains the transcriptional activation domain. Following translocation to the nucleus, p65 binds to specific sequences in the promoter or enhancer regions of many genes associated with cell growth and survival [21]. A large number of tumors have increased NF-κB activity which leads to the constitutive expression of various chemokines and cytokines that stimulate growth and inhibit apoptosis [22]. The expression of pro-inflammatory cytokines can, in turn, stimulate sustained NF-κB activity, forming a positive feedback loop, which may contribute to metastasis [23,24]. Activating the NF-κB pathway also induces changes in the expression of oncogenes, tumor suppressors, and apoptosis inhibitors [25–27].

We show that UCKL-1 over-expression protects tumor cells from NK-mediated and drug-induced apoptosis, enhances tumor survival *in vitro* and *in vivo* and increases the rate of tumor cell proliferation. Over-expression of UCKL-1 increases the expression of cyclin D and cyclin E, which are associated with both cell cycle progression and tumor resistance to apoptosis [28,29]. NF-κB activity is higher in tumor cells transfected with UCKL-1 than in control transfected cells, suggesting at least one possible mechanism by which UCKL-1 influences tumor growth and survival.

## Methods

### Cell culture

K562 human erythroleukemia cells (ATCC, Manassas, VA) and RMA-S mouse lymphoma cells, a gift from Dr. Todd Fehniger, Washington University, St. Louis, MO [30], were cultured in RPMI 1640 media supplemented with 7.5% heat inactivated bovine growth serum (Hyclone, Logan, UT) and 1% L-glutamine. 3T3 (CCL-92) mouse fibroblasts (ATCC) were cultured in DMEM supplemented with 7.5% heat inactivated bovine serum and 1% L-glutamine. RMA-S cells stably expressing green fluorescent protein (GFP) were generated for *in vivo* studies. Cells were transfected with a pcDNA3 vector (Invitrogen, Carlsbad, CA) containing the GFP gene cloned from the pEGFP-N1 vector (Clontech, Mountain View, CA) and selected in media containing 800 μg/mL G418. GFP expression was verified by fluorescence microscopy, flow cytometry, and PCR. Cell counts were determined using a hemocytometer. Cell viability was assessed by trypan blue dye exclusion.

### Transfection with plasmid and siRNA

Transfection by nucleofection was performed using a Nucleofector I device (Lonza/Amaxa Biosystems, Cologne, Germany). Nucleofector kits V, T, and R were used for transfecting K562, RMA-S, and 3T3, respectively, using programs T-16 (K562), A-30 (RMA-S), and U-30 (3T3), according to the manufacturers’ protocols. Cells were transfected with plasmid vector encoding Flag-tagged UCKL-1 (pFlag-UCKL-1) or empty vector, which served as a transfection control (pControl). Over-expression of UCKL-1 in transfected cells was verified by immunoblotting and PCR. The Flag-UCKL-1 plasmid was designed as previously described [13]. The siRNA duplexes were obtained from Invitrogen and resuspended according to the manufacturers’ protocol. K562 cells were nucleofected with 1.5 μM of either siUCKL-1 or sicontrol RNA. Sixteen hours later, the cells were analyzed for down-regulation of UCKL-1 gene expression by quantitative PCR. 3T3 cells were transfected with Flag-tagged UCKL-1 in the pIRES vector in order to generate stable transfectants by selection in G418. Flag-tagged UCKL-1 expression was verified by immunoblotting. Empty vector or sicontrol RNA were used as controls and exhibited no off target effects. Transfection of cells with pFlag-UCKL-1, but not with empty vector, restored cells to their baseline state after siUCKL-1-mediated down-regulation of UCKL-1 [17]. This confirmed the specificity of these reagents.

### Tritiated thymidine proliferation assay

3T3 cells stably transfected with either pIRES (pControl) or pFlag-UCKL-1 pIRES were cultured in serum-deprived media (1% serum) overnight and then plated in normal growth media. After three days in culture, the cells were incubated an additional 16 hours with 4 μCi of tritiated thymidine. Cells were analyzed for tritiated thymidine incorporation as a measure of DNA synthesis. All experiments were performed in triplicate.

### Caspase assay

We measured caspase 3 and 7 activity as indicators of apoptosis. 3T3 cells were nucleofected with pControl or pFlag-UCKL-1 and incubated for 16 hours at 37°C. Staurosporine (12.5 nM) was then added for an additional 6 hours. Caspase activity and cell counts were assessed 22 hours after nucleofection. K562 cells were nucleofected with pControl or pFlag-UCKL-1 and incubated for 16 hours at 37°C. Staurosporine (2.5 μM) or etoposide (50 μM) (Sigma, St. Louis, MO) was then added for an additional 8 hours. Caspase activity was measured 24 hours after nucleofection. Activity was assessed using the Caspase-Glo 3/7 luminescence assay (Promega, Madison, WI), following the manufacturer’s instructions. Triplicate samples were analyzed for luminescence using a microplate reader (Bio-Tek, Winooski, VT). Caspase activity in UCKL-1 transfected cells was calculated relative to control transfected cells.

### Immunoblotting

Immunoblotting was performed to verify UCKL-1 over-expression in UCKL-1 transfected cells and for evaluating levels of cyclin D, cyclin E and β-actin. Cell lysates were run on 8% polyacrylamide gels. Primary antibody anti-Flag M2 along with secondary horseradish peroxidase-conjugated anti-mouse IgG (Sigma) and SuperSignal West Pico Chemiluminescent Substrate (Pierce Biochemicals, Rockford, IL) were used to visualize Flag-tagged UCKL-1. NF-κB p65 antibody and anti-rabbit horseradish peroxidase-conjugated IgG were obtained from Cell Signaling (Danvers, MA). Cyclin D1 and cyclin E1 antibodies was obtained from Santa Cruz Biotechnology (Santa Cruz, CA). β-actin antibody was from Sigma. Bands were analyzed by densitometry using the ChemiDoc XRS+ with Image Lab software (Bio-Rad, Richmond, CA).

### PCR

Endogenous and transfected levels of UCKL-1 mRNA in each cell line were determined by quantitative PCR using UCKL-1 primers (forward primer GTCGCGACGAGTTCATCTC and reverse primer GTCCTCAGGCACGTCGTGGT) that are homologous to both human and mouse UCKL-1. Expression levels were normalized to 18s rRNA. RNA was isolated using the Qiagen RNeasy kit (Qiagen, Valencia, CA) and cDNA prepared using the TaqMan Reverse Transcription Reagents Kit (Applied Biosystems, Foster City, CA). Samples were run on an Applied Biosystems 7500 Real-Time PCR system using iTaq SYBR green supermix (Bio-Rad). Levels of cyclin D1 and cyclin E1 mRNA in control and UCKL-1 transfected cells were similarly determined by quantitative PCR using appropriate mouse or human specific cyclin D1 and E1 primers obtained from Integrated DNA Technologies (Coralville, Iowa).

### NK cell isolation and killing assays

Spleens were harvested from wild type (WT) C57BL/6 (B6) mice. Red blood cells, dead cells and debris were removed by Lympholyte-M density gradient centrifugation (Cedarlane, Ontario, Canada). NK cells were isolated from the mononuclear cell layer using DX5 magnetic beads (Miltenyi Biotec, Bergisch Gladbach, Germany). The DX5+, NK-enriched population was cultured in RPMI 1640 media containing 10% fetal bovine serum, 1% penicillin-streptomycin, 1% L-glutamine and 5 × 10^−5^ M 2-ME in 24 well plates at 4–10 × 10^6^ cells/ml with 1000 U/ml interleukin-2 (IL-2) for 1–3 days before use in cytotoxicity assays.

A fluorescent, calcein labeled tumor killing assay was modified from Roden et al. [31]. Briefly, UCKL-1 or control transfected RMA-S cells (5 × 10^5^) were labeled with 5 μM calcein at 37°C for 30 minutes. DX5 + NK cells were then cultured with transfected RMA-S cells for four hours at 37°C at effector to target cell ratios of 100:1 and 50:1. Controls include a media control, a maximum release control (tumor cells lysed in a 2% Triton X-100 detergent solution), and a spontaneous release control (tumor cells incubated without NK cells). Values were calculated as described by Roden et al., in which the percent specific lysis is the amount of calcein released by tumor cells incubated with NK cells relative to the maximum calcein released by detergent lysis of tumor cells [31]. NK lytic index is defined as the percentage of tumor cells lysed, with the killing of control transfected cells set to 100%.

### RMA-S-GFP tail vein injections

RMA-S-GFP cells were nucleofected with pControl or pFlag-UCKL-1 and harvested 24 hours later. Control or UCKL-1 transfected RMA-S-GFP cells (5 × 10^5^) were injected into the tail vein of WT B6 and NKLAM-deficient knockout (KO) B6 mice. Four hours after injection, mice were sacrificed and their lungs were harvested. The azygous lobe of the lung was processed from each mouse as previously described [32]. Lung RNA was prepared with the Qiagen RNeasy kit and cDNA was reverse transcribed using the TaqMan Reverse Transcription Reagents Kit. Samples were run on a 7500 Applied Biosystems Real-Time PCR system using Taqman primer/probes for GFP and 18s rRNA as previously described [32]. Change in Ct (ΔCt) was calculated with 18S rRNA as a housekeeping gene control. The level of GFP expression is a direct measure of tumor burden in the lung [32]. For each experiment, over-expression of UCKL-1 in UCKL-1 transfected RMA-S-GFP cells was confirmed by immunoblotting.

### NF-κB activity

K562 cells were nucleofected with the NF-κB luciferase reporter plasmid (pNF-κB-Luc Vector; Clontech) and pFlag-UCKL-1, pControl, or siRNA (siUCKL-1 or sicontrol RNA). Cells were also co-transfected with the pSV-β-galactosidase (β-gal) control vector (Promega) for determination of transfection efficiency. NF-κB activity was assessed using the Luciferase Assay and β-gal activity was assessed using the β-Galactosidase Enzyme Assay, per the manufacturers’ protocol (Promega). Protein concentrations were determined with a BCA protein assay (Pierce). NF-κB readings were normalized to β-gal activity and protein levels in all experiments.

### Nuclear and cytoplasmic extracts

Nuclear and cytoplasmic extracts from K562 cells nucleofected with pControl or pFlag-UCKL-1 were prepared 24 hours after nucleofection using NE-PER Nuclear and Cytoplasmic Extraction Reagents (Pierce). Nuclear and cytoplasmic lysates were run on gels and immunoblots were probed for NF-κB p65. The purity of the extracts was assessed using antibodies to tubulin (Sigma) and lamin A/C (Santa Cruz Biotechnology). BCA assays were run to ensure equal protein loading of extracts. Levels of IκB in the cytoplasm after nucleofection were assessed by immunoblot analysis using an IκB antibody (Santa Cruz Biotechnology). As a positive control for IκB degradation, cytoplasmic extracts from K562 cells treated with tumor necrosis factor (TNF)-α (5 ng/ml) for 30 minutes at 37°C were immunoblotted for IκB.

## Statistical Analysis

A Student’s *t*-test was used for all statistical analyses with *p* ≤ 0.05 deemed significant.

## Results

### UCKL-1 over-expression in transfected cells

**Figure 1:**
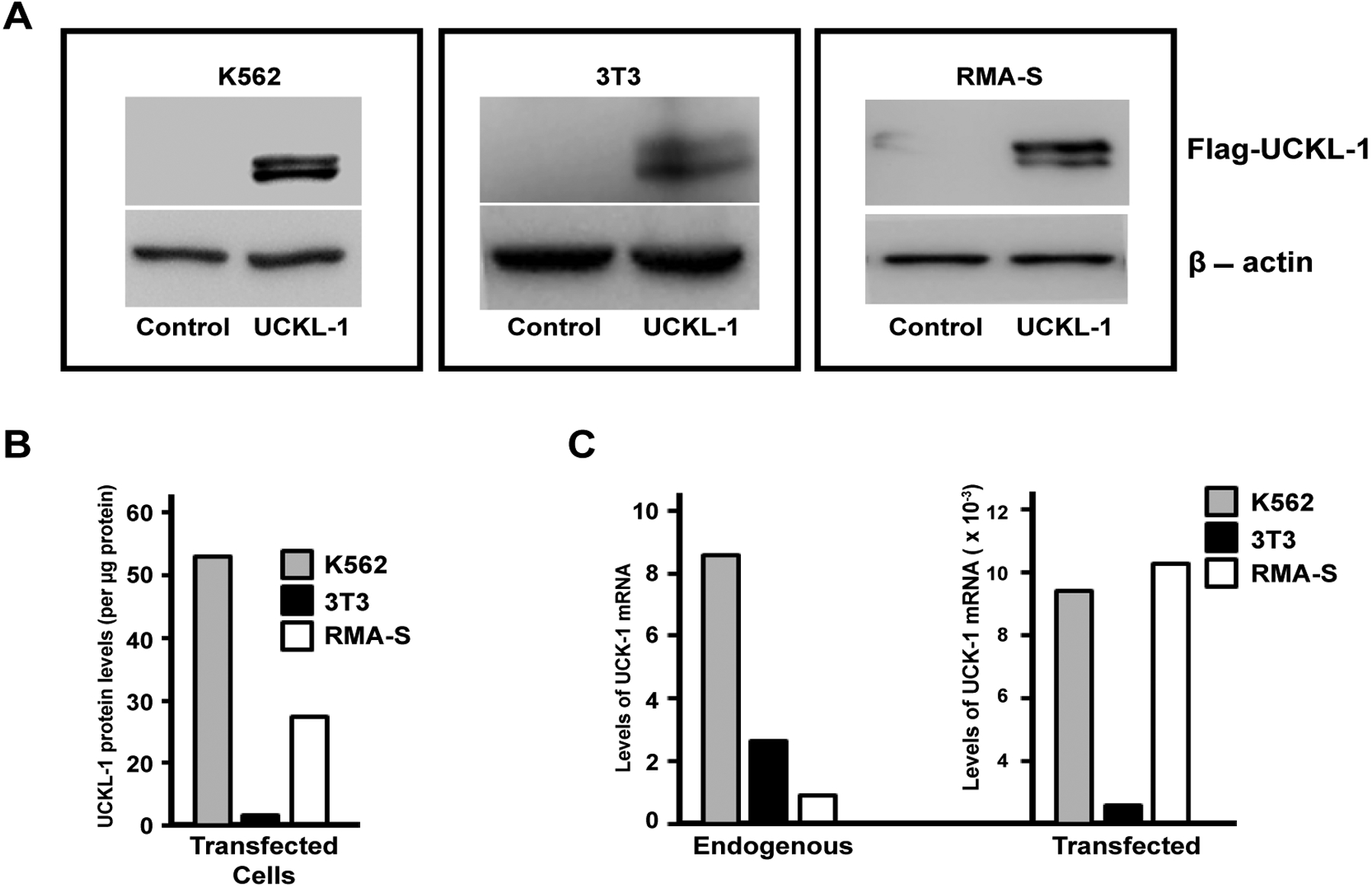
UCKL-1 over-expression in transfected cell lines. (**a**) Immunoblot analysis of pControl and pFlag-UCKL-1 transfected K562, 3T3 and RMA-S cells was performed. The expression of Flag illustrates over-expression of UCKL-1 protein in pFlag-UCKL-1 transfected cells. β-actin was used as a loading control; (**b**) Levels of Flag-tagged UCKL-1 in transfected K562, 3T3 and RMA-S cells were compared by densitometry of immunoblots. Relative levels of Flag-UCKL-1 in each cell line are depicted. Flag-tagged UCKL-1 protein expression was lowest in UCKL-1 transfected 3T3 cells, which was set to a value of one; (**c**) Quantitative PCR was performed for detection of UCKL-1 mRNA in UCKL-1 and control transfected K562, 3T3 and RMA-S cells. 18s rRNA was the housekeeping gene control. The lowest endogenous level of UCKL-1 mRNA was found in RMA-S, and was set to a value of one (left panel). There was a 1,000 fold increase in UCKL-1 mRNA in UCKL-1 transfected 3T3 cells, and approximately 10,000 fold increases in both UCKL-1 transfected K562 and RMA-S cells (right panel).

Over-expression of UCKL-1 was achieved by transfection using nucleofection technology. Tumor cell lines K562 and RMA-S and the mouse fibroblast cell line 3T3 were transfected with an empty plasmid vector (pControl) or plasmid encoding Flag-tagged UCKL-1 (pFlag-UCKL-1). Protein expression was verified by immunoblotting. Flag-tagged UCKL-1 runs as a doublet of approximately 55–60 kD, which is consistent with its predicted molecular weight (Figure 1a). The relative levels of Flag-UCKL-1 protein in each transfected cell line are depicted in figure 1b. K562 expresses 53 times more Flag-tagged UCKL-1 than transfected 3T3 cells, while RMA-S expresses 27 times more UCKL-1 than transfected 3T3 cells. Expression of UCKL-1 mRNA was quantitated by PCR (Figure 1c). Endogenous levels of UCKL-1 mRNA in K562 are eight fold higher than in RMA-S cells and three fold higher than in 3T3 cells. After transfection with pFlag-UCKL-1, there is a 1,000 fold increase in UCKL-1 mRNA in 3T3 cells and approximately 10,000 fold increases in UCKL-1 mRNA levels in both K562 and RMA-S. The levels of UCKL-1 mRNA are consistent with the levels of protein expressed after transfection. Peak UCKL-1 protein over-expression was seen approximately 24 hours after nucleofection in all three cell lines.

### Effect of UCKL-1 over-expression on apoptosis in the K562 human erythroleukemia cell line and in 3T3 mouse fibroblasts

**Figure 2:**
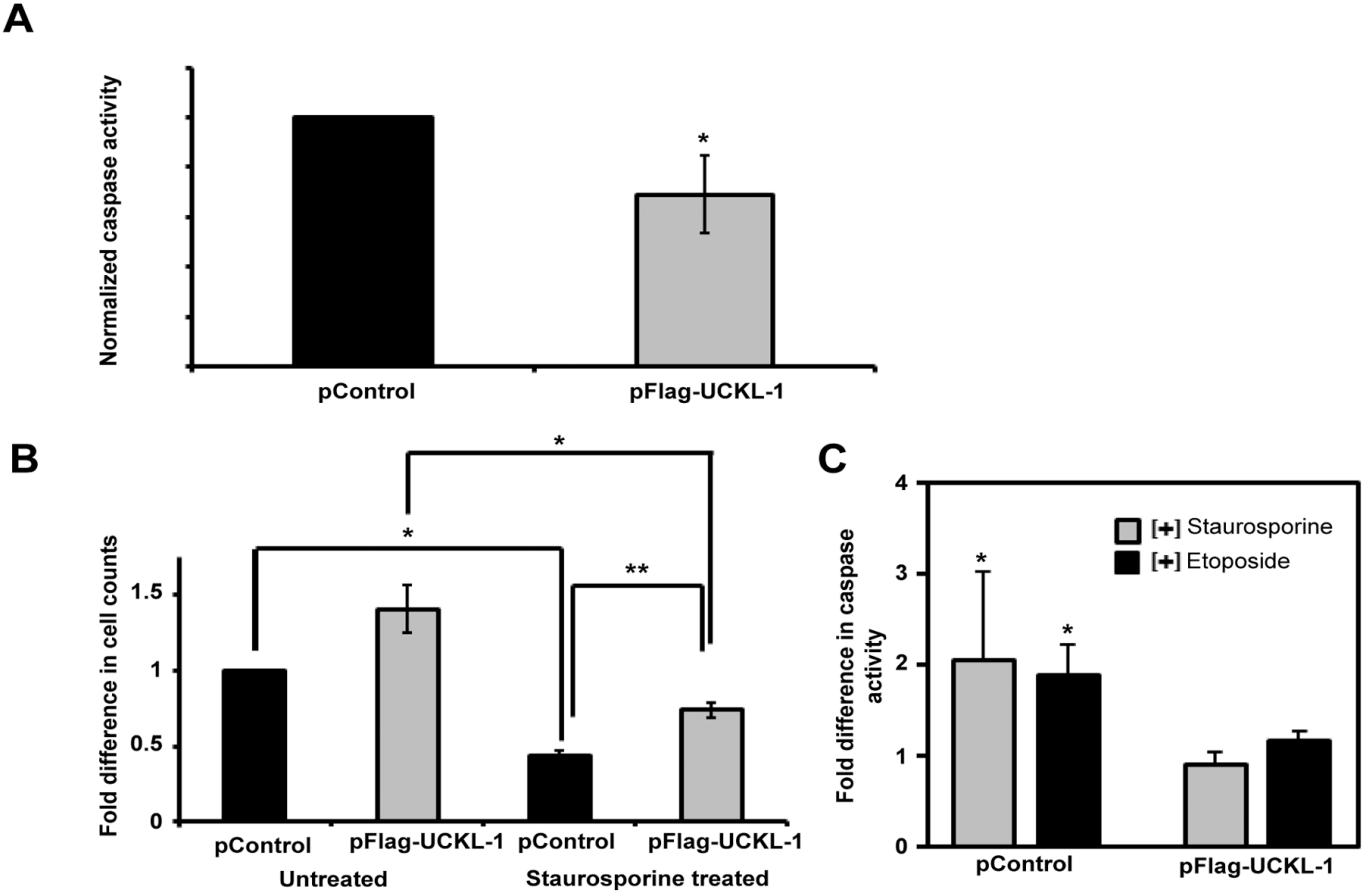
Effect of UCKL-1 over-expression on apoptosis in K562 cells. Caspase 3/7 activity was measured 24 hours after nucleofection of K562 cells with either pFlag-UCKL-1 or pControl. Data are a summary of five replicate experiments. (**a**) Spontaneous apoptosis of transfected K562 cells was evaluated. Significantly less caspase activity was observed in UCKL-1 transfected than control transfected K562 cells (*p = 0.03); (**b** & **c)** Staurosporine (2.5 μM) was added to transfected K562 cells to induce apoptosis. (**b**) Cells were counted 24 hours after transfection and the counts were normalized to the level of untreated control transfected cells (pControl) (*p = 0.03, **p = 0.002); (**c**) Caspase activity was lower in staurosporine and etoposide (50 μM) treated UCKL-1 transfected K562 cells than in similarly treated control transfected cells (*p = 0.02).

Apoptosis was measured by caspase 3/7 activity. The level of spontaneous apoptosis in UCKL-1 transfected K562 cells was 30% less than in control-transfected cells (Figure 2a). K562 cells over-expressing UCKL-1 were also more resistant to apoptosis induced by the chemotherapeutic drugs staurosporine and etoposide than control cells (Figure 2b and Figure 2c). UCKL-1 transfected, staurosporine-treated cultures showed significantly higher cell counts and lower caspase activity than staurosporine-treated control transfected cultures (Figure 2b and Figure 2c).

**Figure 3:**
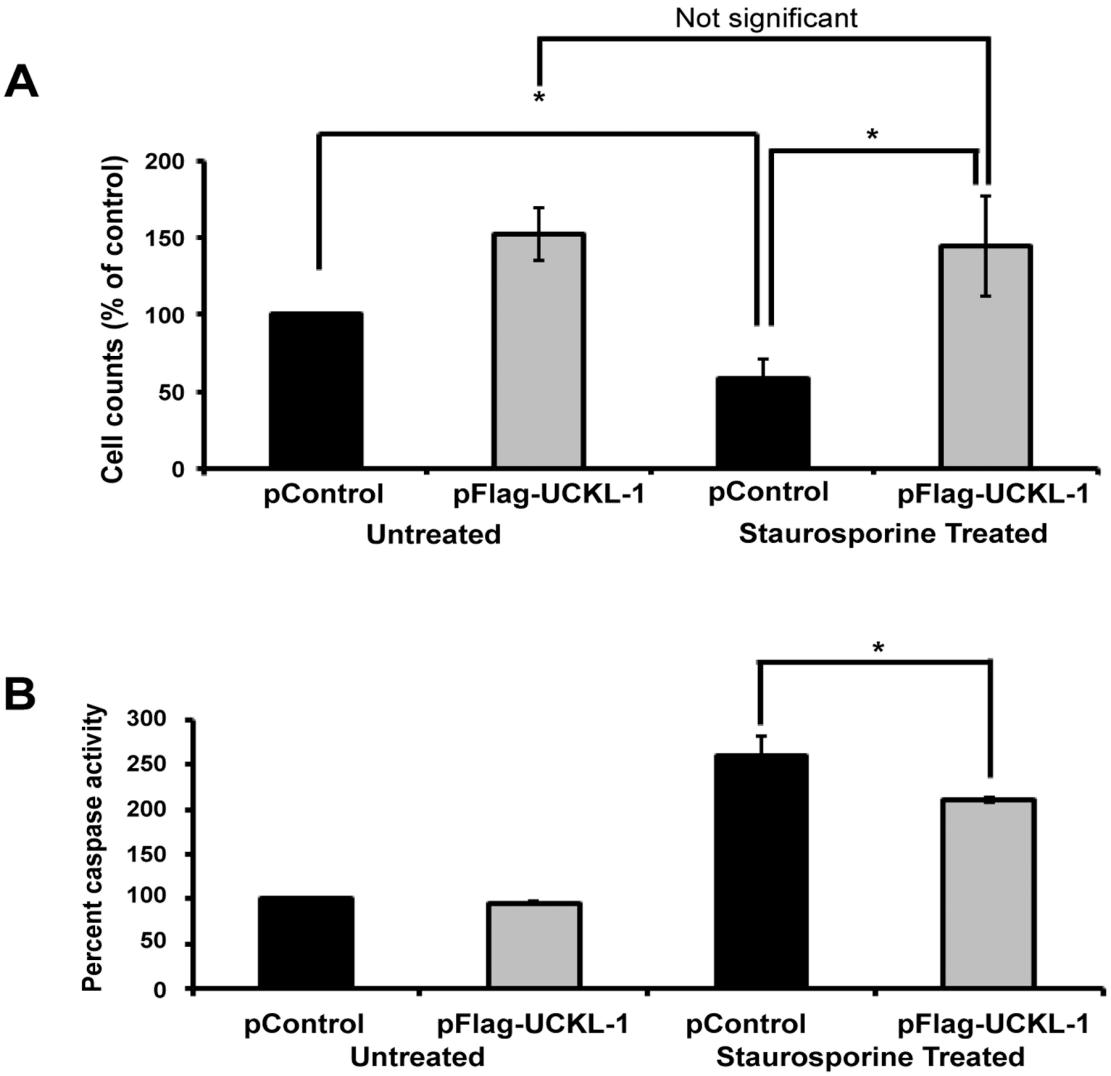
Effect of UCKL-1 over-expression on apoptosis induction in the 3T3 mouse fibroblast cell line. Staurosporine (12.5 nM) was added to control and UCKL-1 transfected 3T3 cells to induce apoptosis. Data are a summary of six replicate experiments. (**a**) Cell counts 22 hours after transfection were normalized to the level of untreated control transfected cells (pControl) (*p = 0.02). (**b**) UCKL-1 over-expression protected 3T3 cells from staurosporine-induced apoptosis. Caspase activity was measured 22 hours after nucleofection. Activity was normalized to untreated pControl-transfected cells, which was set to 100 (*p = 0.01).

To determine if UCKL-1 functions similarly in non-cancer cells, 3T3 mouse fibroblasts were evaluated. These cells have lower levels of endogenous UCKL-1 than K562. 3T3 cells transfected with UCKL-1 had a significantly higher cell count than control-transfected cells (Figure 3a). They were also significantly less susceptible to staurosporine-induced apoptosis than control transfected 3T3 cells (Figure 3a). Significantly less caspase activity was also observed in UCKL-1-transfected, staurosporine-treated 3T3 cells than in staurosporine-treated control transfected 3T3 cells (Figure 3b). These results indicate that high UCKL-1 levels provide protection from apoptosis induced by chemotherapeutic agents in both K562 tumor cells and 3T3 fibroblasts. The level of spontaneous apoptosis in 3T3 cells is low; therefore, no measurable difference in caspase activity between untreated control and UCKL-1-transfected 3T3 cells was detected (Figure 3b).

### Lower susceptibility of RMA-S cells over-expressing UCKL-1 to NK-mediated killing *in vitro* and *in vivo*

**Figure 4:**
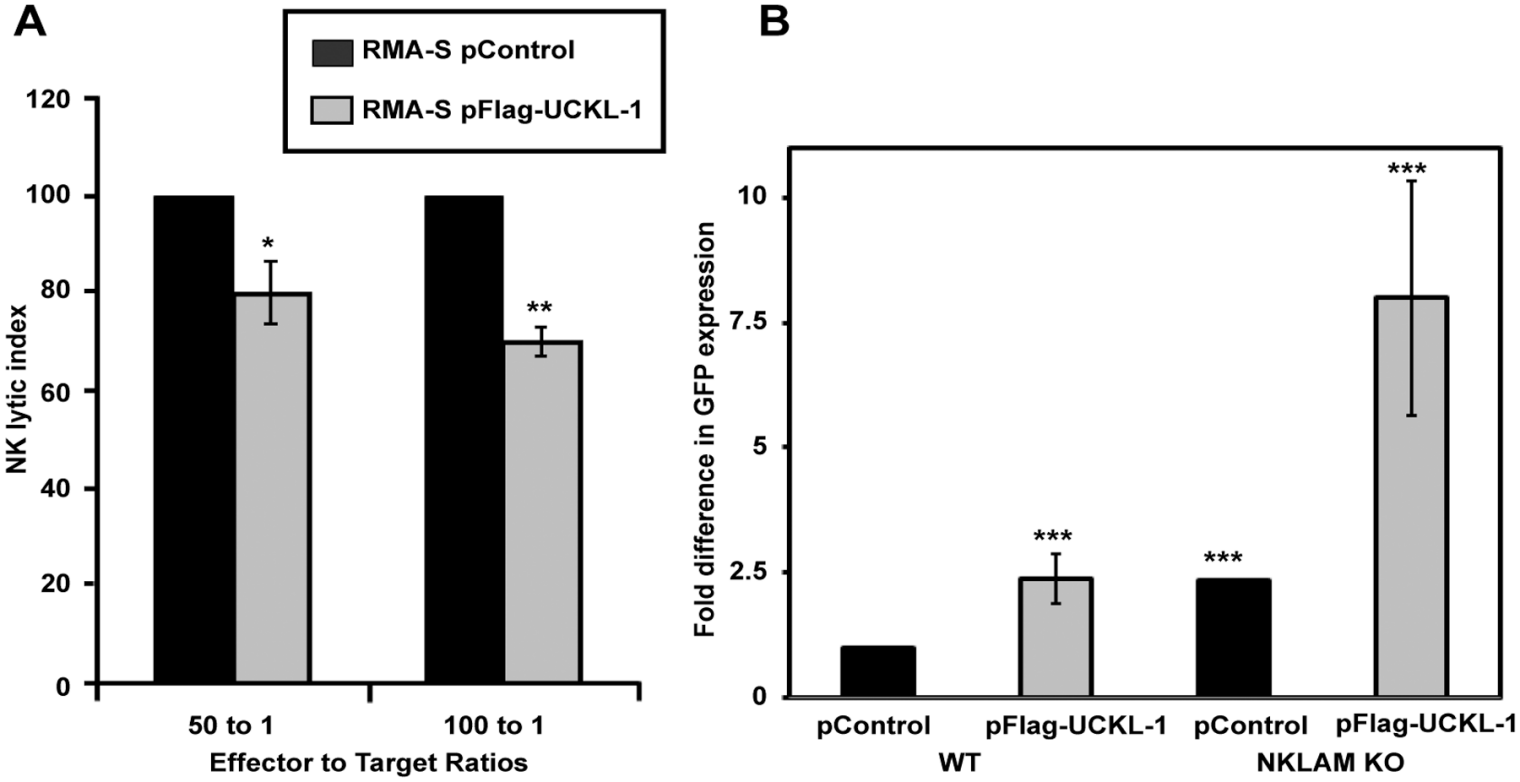
Decreased susceptibility of RMA-S cells over-expressing UCKL-1 to NK killing *in vitro* and *in vivo*. (**a**) RMA-S cells transfected with pControl or pFlag-UCKL-1 were incubated with WT NK cells for four hours. UCKL-1 transfected cells were significantly more resistant to NK lysis at both effector to target cell ratios of 100 to 1. Data are a summary of four replicate experiments. (*p = 0.04) and 50 to 1 (**p = 0.03). NK lytic index represents tumor killing, with the lysis of control transfected cells set to 100% in each experiment. (**b**) RMA-S cells stably expressing GFP (RMA-S-GFP) were nucleofected with either pControl or pFlag-UCKL-1 and injected into the tail vein of WT or NKLAM KO mice 24 hours after transfection. Mice were sacrificed four hours later and the lungs were harvested. The level of GFP in the lung, reflecting the number of surviving RMA-S-GFP cells, was quantitated by real-time PCR. A significantly greater number of UCKL-1 transfected RMA-S-GFP cells than control-transfected cells survived in the lungs of WT mice (RMA-S-GFP pControl: n = 15, RMA-S-GFP pFlag-UCKL-1, n = 16). The increase in tumor survival was potentiated in NKLAM KO mice (RMA-S-GFP pControl: n = 4, RMA-S-GFP pFlag-UCKL-1: n = 5) (***p = 0.03). Fold differences in GFP expression were relative to the level of GFP in the lungs of WT mice injected with pControl-transfected RMA-S-GFP cells.

We evaluated whether UCKL-1 over-expression would protect tumor cells from NK-mediated killing through induction of apoptosis. The NK susceptible mouse lymphoma cell line RMA-S was used for these experiments. Control and UCKL-1-transfected RMA-S cells were co-cultured with purified NK cells for four hours *in vitro*. UCKL-1 transfected RMA-S cells were significantly more resistant to NK killing than control transfected RMA-S cells (Figure 4a). Experiments were conducted to determine the influence of UCKL-1 levels on the survival of NK sensitive RMA-S cells *in vivo*. Injection of NK-sensitive tumor cells into the tail vein of mice, followed by quantitation of surviving tumor cells in the lungs four hours later, is a well described model of NK-mediated killing *in vivo* [33]. RMA-S cells stably expressing GFP (RMA-S-GFP) were used in these *in vivo* experiments, where GFP serves as a tumor-specific marker. RMA-S-GFP cells were transfected with either pControl or pFlag-UCKL-1.

Twenty four hours later, transfected cells were injected into the tail vein of wild type (WT) B6 or NKLAM-deficient knockout (KO) mice. Lungs were harvested four hours after cell injection and analyzed for tumor cell levels. Real-time PCR using a GFP primer/probe was performed as a quantitative measure of RMA-S-GFP tumor cell survival in the lungs. More than twice as many UCKL-1 transfected tumor cells than control-transfected cells survived in the lungs of WT mice (Figure 4b). The increased cellular persistence of UCKL-1-transfected RMA-S-GFP cells was potentiated in NKLAM KO mice. NKLAM KO mice injected with UCKL-1 transfected RMA-S-GFP cells had approximately eight fold more lung GFP expression than WT mice injected with control transfected RMA-S-GFP cells. The higher GFP expression indicates a greater number of tumor cells, hence more tumor survival in the lung (Figure 4b). Tumor burden was greatest in NKLAM KO mice injected with UCKL-1 transfected tumor cells than in any other group, indicating that UCKL-1 over-expression protects tumor cells from immune-mediated destruction *in vivo*.

### Effect of UCKL-1 over-expression on proliferation of 3T3 cells *in vitro*

**Figure 5:**
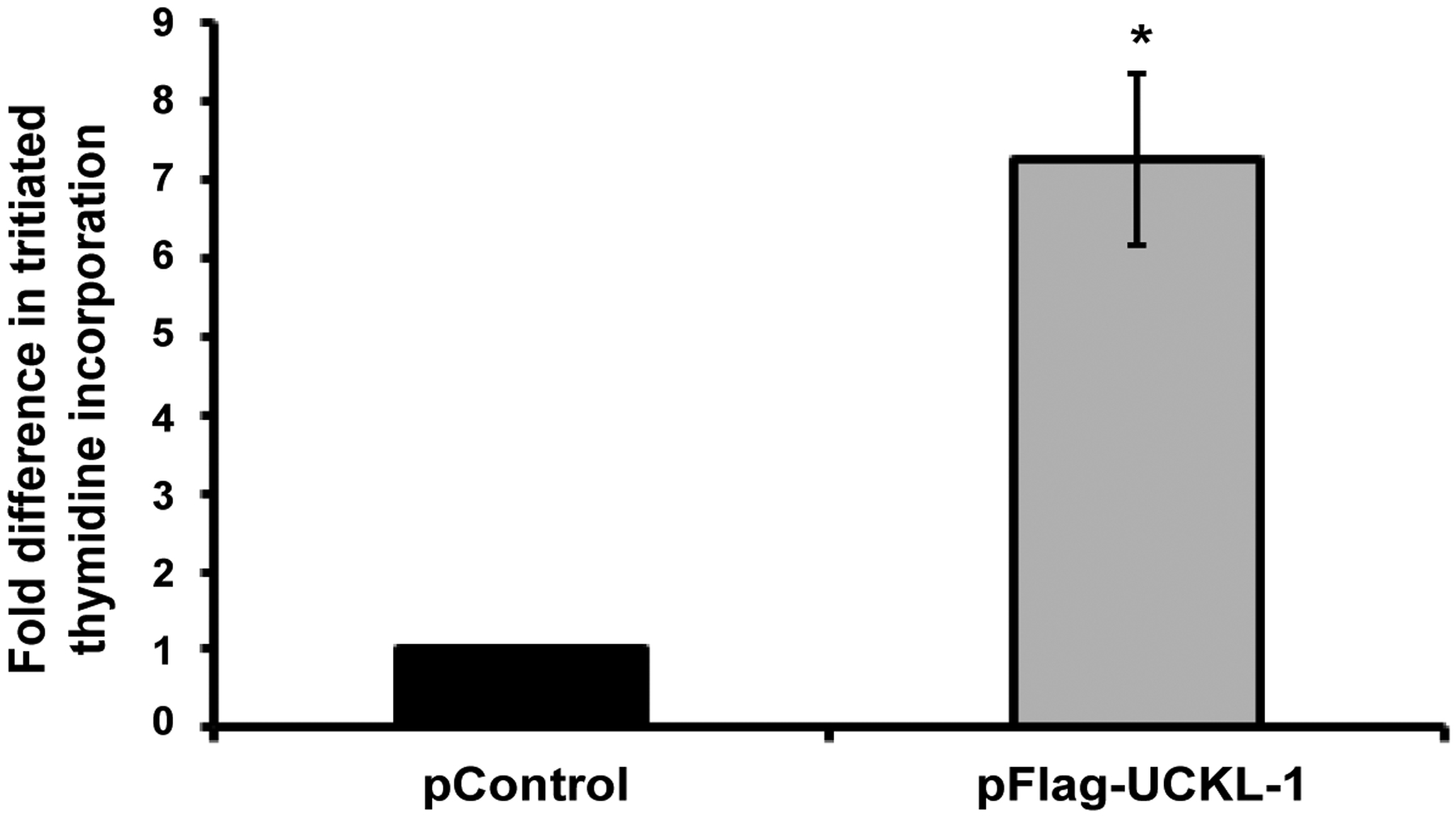
Effect of UCKL-1 over-expression on proliferation of 3T3 cells. 3T3 cells stably expressing Flag-tagged UCKL-1 or empty vector (pControl) were analyzed for tritiated thymidine incorporation. UCKL-1 transfected cells incorporated significantly more tritiated thymidine than control transfected cells, indicating more DNA synthesis (*p = 0.02). Data are a summary of three replicate experiments.

We generated a stable line of 3T3 mouse fibroblasts over-expressing UCKL-1 and used tritiated thymidine incorporation assays to quantitate proliferation. UCKL-1 transfected 3T3 cells have a significantly higher proliferation rate than empty vector transfected 3T3 cells (Figure 5).

### Effect of UCKL-1 over-expression on cyclin D and cyclin E levels in K562 and 3T3 cells

Cyclins D and E play a critical role in cell cycle progression and induction of proliferation. Over-expression of cyclin E shortens the G1 phase and prolongs the S phase; cells must reach a threshold level of cyclin E expression to proceed into S phase [28,34]. Cyclin D synthesis is also initiated during the G1 phase and acts along with cyclin E at the G1 to S transition to promote cell proliferation [29].

**Figure 6:**
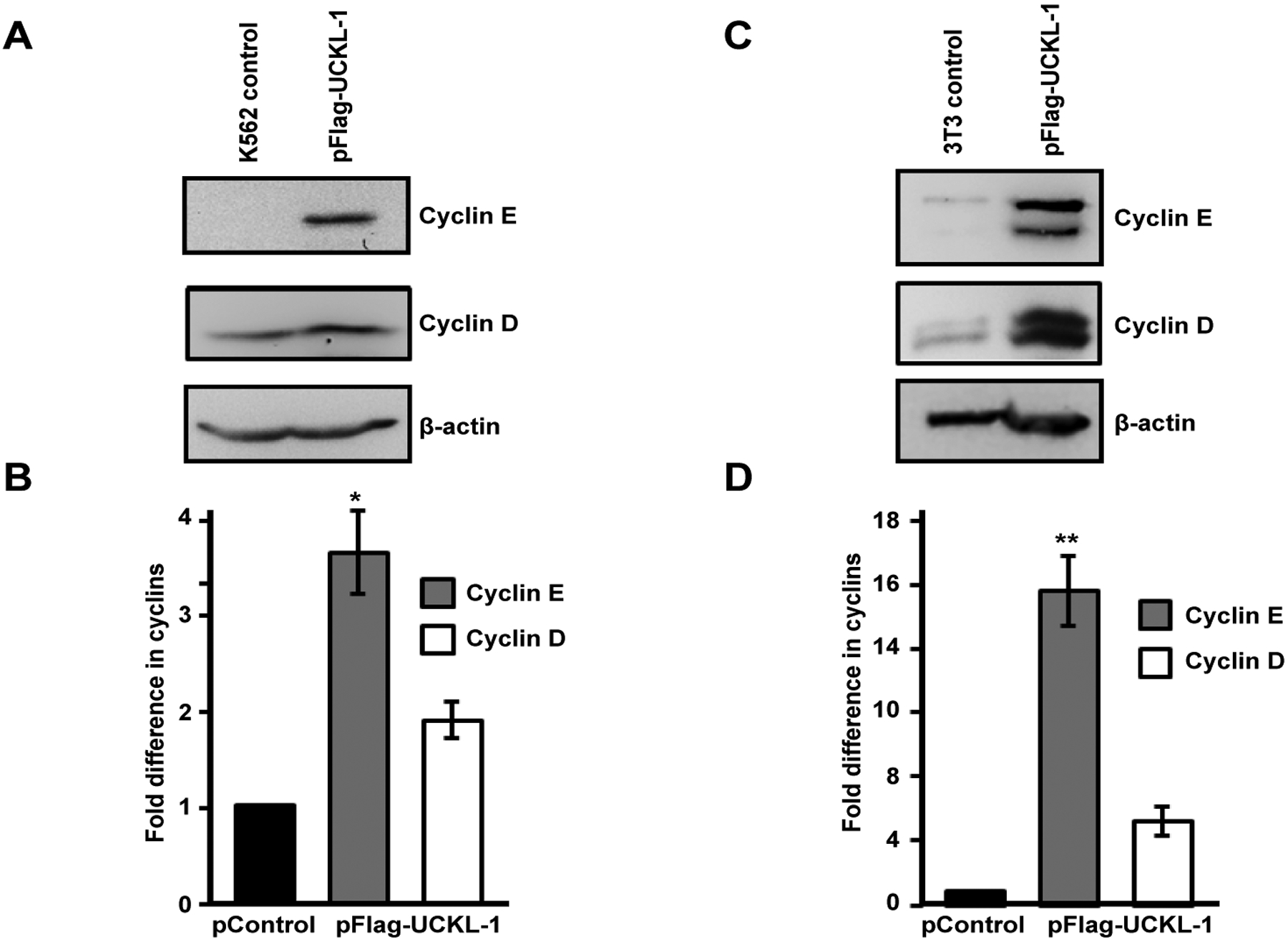
Effect of UCKL-1 over-expression on cyclin D and cyclin E levels. **(a** & **b)** K562 cells were nucleofected with either pFlag-UCKL-1 or pControl and cell lysates were analyzed for cyclin D, cyclin E and β-actin expression 20 hours later. A representative blot is shown in (**a**); (**b**) Cyclin D and cyclin E levels in K562 cells were quantitated from immunoblots from four replicate experiments. Bands were analyzed by densitometry and cyclin levels were normalized to β-actin. Results are expressed as the fold increase in cyclin expression, with the levels in pControl-transfected K562 cells set to one (*p = 0.002); (**c** & **d)** Cell lysates from 3T3 cells transfected with pFlag-UCKL-1 or pControl were analyzed for cyclin D, cyclin E and β-actin expression. A representative blot is shown in (**c**); (**d**) Cyclin D and cyclin E levels were quantitated from immunoblots from three experiments. Bands were analyzed by densitometry and cyclin levels were normalized to β-actin. Results are expressed as the fold increase in cyclin expression, with the levels in pControl-transfected 3T3 cells set to one (**p = 0.006).

Significantly higher levels of cyclin D and cyclin E were found in UCKL-1 transfected K562 cells 20 hours after nucleofection than in control transfected cells (Figure 6a and Figure 6b). Four replicate experiments were performed with similar results.

Significantly higher levels of cyclin D and cyclin E were also observed in UCKL-1 transfected 3T3 cells than in control transfected cells (Figure 6c and Figure 6d). This effect was even more dramatic in 3T3 cells than in K562 cells. Both cyclin D and cyclin E run as doublets on immunoblots of 3T3 lysates, which is consistent with previous published reports [35,36]. Three replicate experiments were performed with similar results.

Levels of cyclin D1 and cyclin E1 mRNA were assessed by quantitative PCR and were consistently higher in UCKL-1 transfected cells than in control transfected cells. There was 1.6 fold more cyclin E and 1.3 fold more cyclin D mRNA in UCKL-1 transfected K562 cells than in control-transfected cells. Similarly, in UCKL-1 transfected 3T3 cells, there was 2.4 fold more cyclin E mRNA and 2.6 fold more cyclin D mRNA than in control-transfected cells.

### Effect of UCKL-1 expression on NF-κB activity in K562 cells

**Figure 7:**
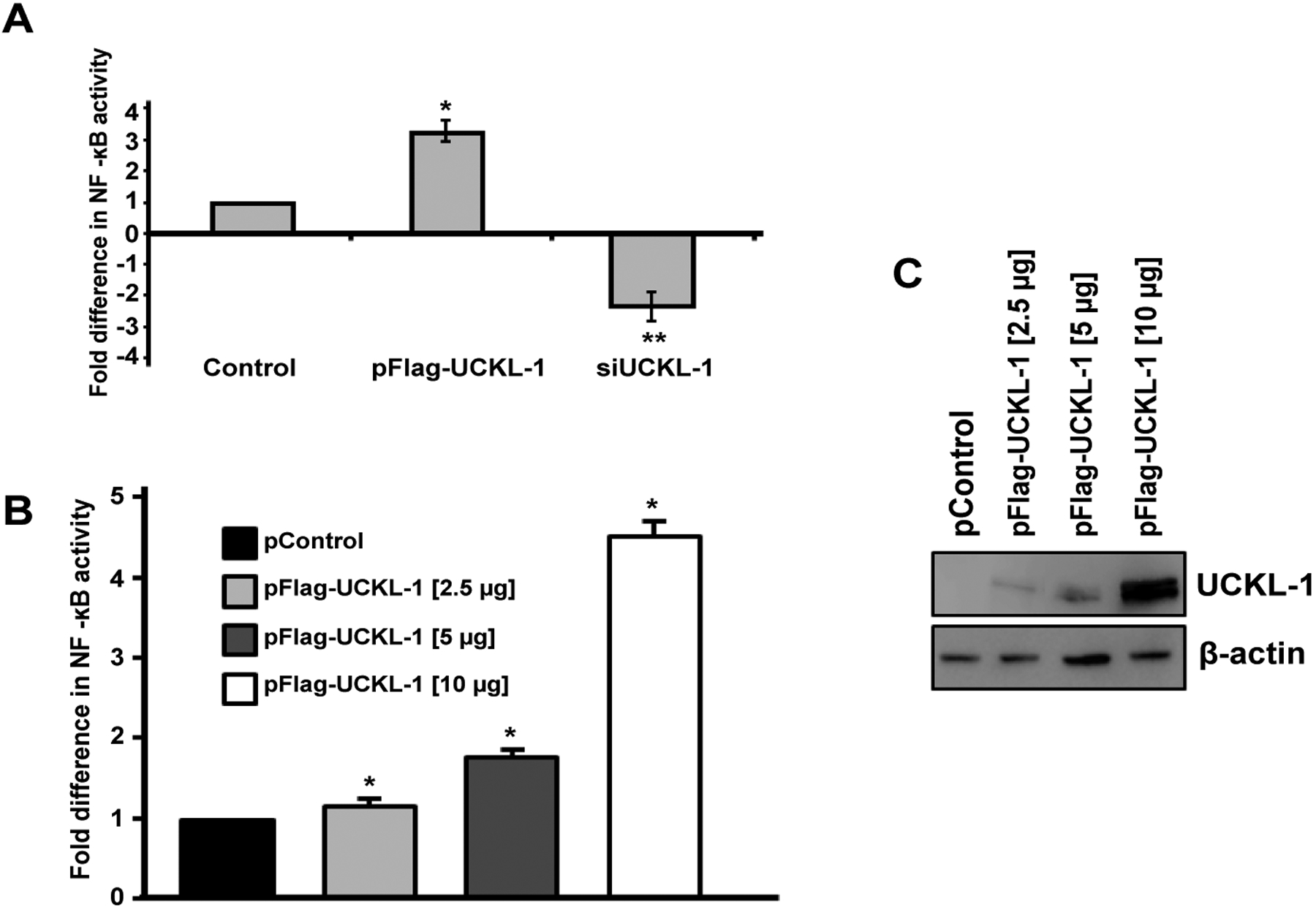
Effect of UCKL-1 levels on NF-κB activity in K562 cells. NF-κB activity was measured by transfection of an NF-κB luciferase expression plasmid. (**a**) Significantly higher levels of NF-κB activity were observed in UCKL-1 transfected than in control transfected cells (*p = 0.02). Significantly less NF-κB activity was present in siUCKL-1-treated cells compared to sicontrol-treated cells (**p = 0.04). Data are a summary of four replicate experiments. (**b** & **c**) UCKL-1 increases NF-κB activity in a dose-dependent manner. K562 cells were nucleofected with the NF-κB luciferase reporter plasmid along with 2.5, 5 or 10 μg pFlag-UCKL-1 or 10 μg pControl; (**b**) NF-κB activity and c UCKL-1 protein levels, were analyzed 24 hours later. There is a dose-dependent increase in NF-κB activity with increasing levels of UCKL-1 (*p < 0.01). Data are a summary of five replicate experiments.

**Figure 8:**
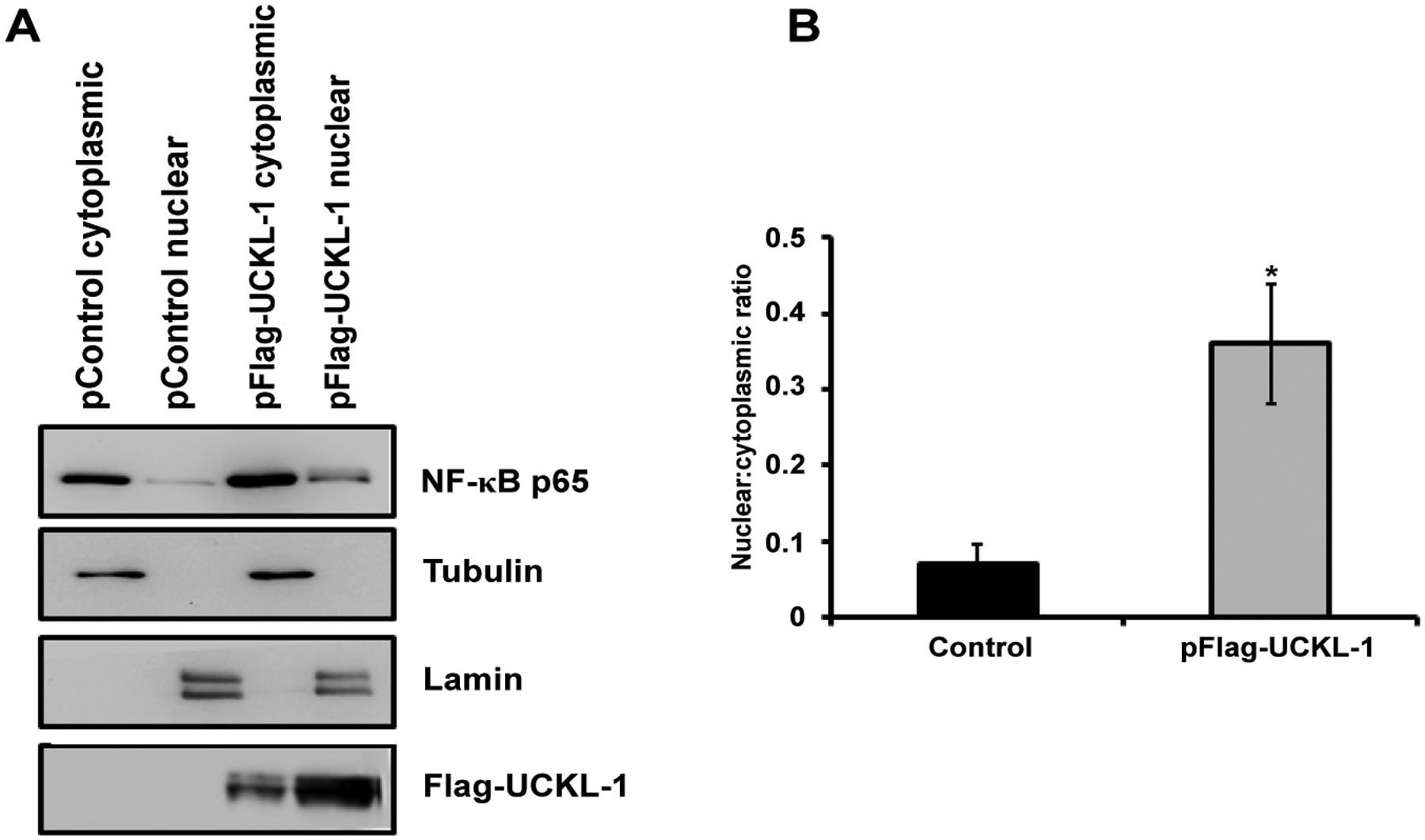
Nuclear and cytoplasmic NF-κB p65 levels in extracts from control and UCKL-1 transfected K562 cells were determined by immunoblotting. (**a**) A representative immunoblot demonstrating more translocation of p65 to the nucleus in UCKL-1 transfected K562 cells is shown. Extracts were assessed for nuclear purity by expression of lamin. The purity of cytoplasmic extracts was verified by tubulin levels. Results indicate minimal cross-contamination of nuclear and cytoplasmic extracts; (**b**) Quantization of NF-κB p65 illustrates that five times more NF-κB p65 translocated to the nucleus in UCKL-1 transfected K562 cells compared to control transfected cells. Data are a summary of three experiments (*p = 0.05).

As a first step towards identifying a potential mechanism by which UCKL-1 functions, we evaluated the effect of UCKL-1 levels on NF-κB activity. As a transcriptional activator, NF-κB plays a role in many of the cellular activities influenced by UCKL-1, including apoptosis, proliferation and immune functions. To measure NF-κB activity, an NF-κB luciferase reporter plasmid was co-transfected with pControl or pFlag-UCKL-1 plasmids into K562 cells. Endogenous NF-κB proteins bind to the κ enhancer element in the reporter plasmid, inducing luciferase transcription. The amount of luminescence released is a measure of NF-κB activity. NF-κB readings were normalized to both β-gal activity (to control for transfection efficiency) and to protein levels. UCKL-1 transfected K562 cells had significantly higher NF-κB activity than control transfected cells; siUCKL-1 treated K562 cells had less NF-κB activity (Figure 7a). K562 cells were transfected with different concentrations of pFlag-UCKL-1 plasmid and NF-κB activity and UCKL-1 protein levels determined. NF-κB activity directly correlates with the level of UCKL-1 expression, indicating that UCKL-1 activates NF-κB in a dose-dependent manner (Figure 7b and Figure 7c). NF-κB must translocate from the cytoplasm to the nucleus to function. Therefore, translocation of the NF-κB transcriptional activation subunit p65 from the cytoplasm to the nucleus was examined. Nuclear and cytoplasmic fractions of control and UCKL-1 transfected K562 cells were prepared and examined for levels of NF-κB p65 by immunoblotting. Significantly more NF-κB p65 was translocated to the nucleus in UCKL-1 transfected cells than in control-transfected cells (Figure 8a and Figure 8b). The purity of the nuclear and cytoplasmic extracts was verified by probing the blots with cytoplasmic protein tubulin and nuclear protein lamin. Minimal cross-contamination of the fractions was seen (Figure 8a). There is 2.6 fold more UCKL-1 retained in the nucleus of UCKL-1 transfected cells, with less detected in the cytoplasm (Figure 8).

**Figure 9:**
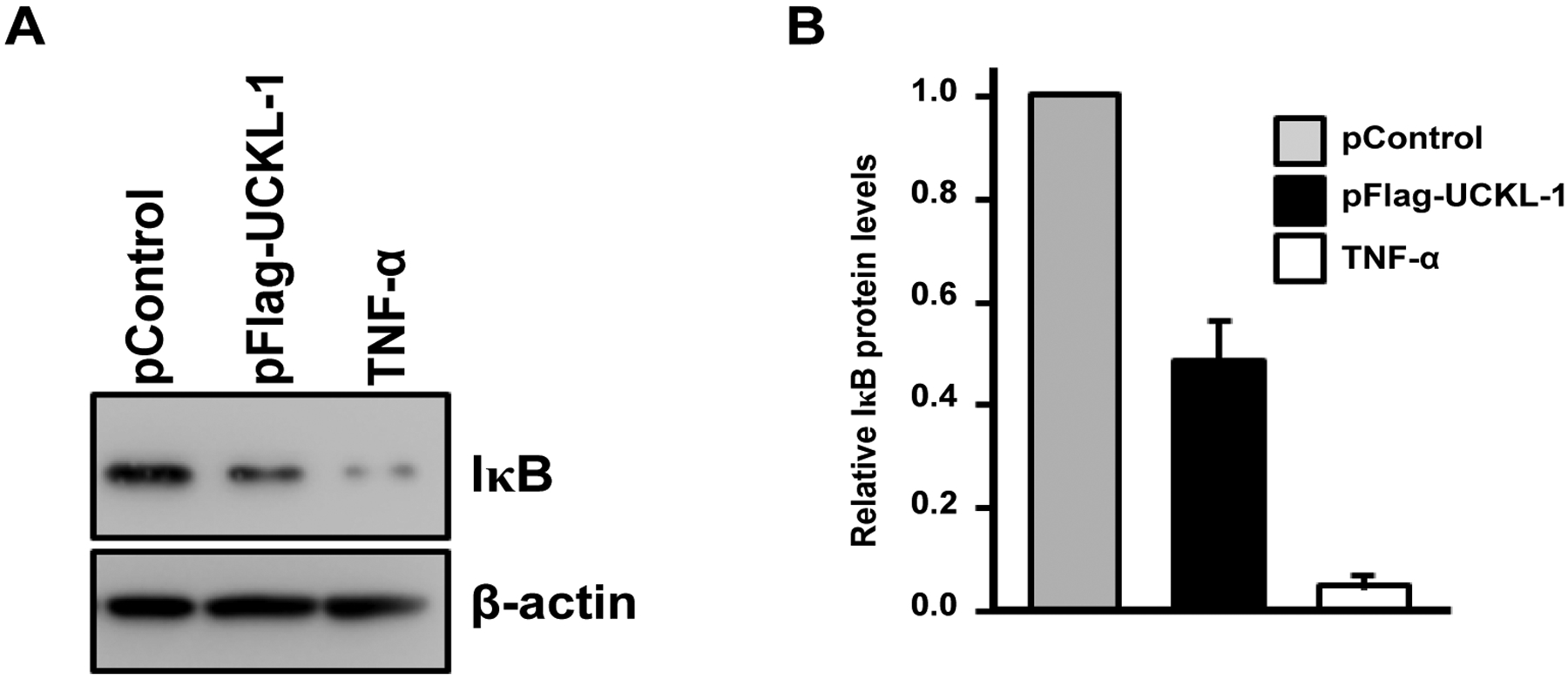
Effect of UCKL-1 over-expression on IκB levels in the cytoplasm. Cytoplasmic extracts from pControl or pFlag-UCKL-1 transfected K562 cells were analyzed for levels of IκB 20 hours after nucleofection by immunoblotting. Extracts from K562 cells stimulated with TNF-α (5 ng/ml) for 30 minutes were run as a positive control. (**a**) A representative blot is shown; (**b**) Quantization of IκB illustrates that there is significantly less IκB in the cytoplasm of UCKL-1 transfected cells than in control-transfected cells. Data are a summary of four experiments (*p ≤ 0.02).

IκBα, an inhibitory subunit in the NF-κB complex, plays a major role in controlling the nuclear translocation of NF-κB. IκBα is phosphorylated, ubiquitinated, and then degraded, which releases NF-κB to translocate to the nucleus [20,21]. We therefore compared the level of IκBα and its degradation in both UCKL-1 and in control-transfected cells. As a positive control, K562 cells were stimulated with TNF-α for 30 minutes to induce IκB degradation. There is 50% less IκB in the cytoplasm of UCKL-1 transfected K562 cells than in control-transfected cells, suggesting more degradation of IκB occurs with high levels of UCKL-1 expression (Figure 9).

## Discussion

We previously identified UCKL-1 as a substrate for ubiquitination by the E3 ligase NKLAM [12]. High levels of NKLAM are associated with enhanced tumor killing. In this study, we examined the functional consequence of over-expressing UCKL-1 as a means to begin to identify its mechanism of action. We evaluated the effect of UCKL-1 over-expression on susceptibility of K562 and 3T3 cells to apoptosis mediated by NK cells and chemotherapeutic drugs. Over-expression of UCKL-1 resulted in significantly greater resistance of both K562 and 3T3 cells to staurosporine-induced apoptosis. UCKL-1-transfected K562 cells were also more resistant to induction of apoptosis by etoposide. This suggests that increasing the level of UCKL-1 protects cells from drug-induced death.

The immune system is an important component of the innate and cognate defense against tumor initiation and progression. The lymphoma line RMA-S is a prototypic NK tumor target and therefore a good model for evaluating the effect of UCKL-1 over-expression on tumor susceptibility to NK killing.

*In vitro*, UCKL-1 transfected RMA-S cells were significantly more resistant to NK lysis than control transfected cells. Similar results were also observed *in vivo*. More UCKL-1 transfected RMA-S-GFP cells persisted in the lungs of WT and NKLAM KO mice than control-transfected RMA-S-GFP cells after intravenous administration of tumor cells. Tumor burden was greatest in NKLAM KO mice injected with UCKL-1 transfected RMA-S cells than in any other group. These results are consistent with data showing that NKLAM plays a critical role in tumor cell lysis, at least in part, by ubiquitinating UCKL-1, leading to its degradation in the tumor target cell [17]. The absence of NKLAM in the host results in even less tumor killing. The observation that there is some tumor killing in NKLAM KO mice suggests that there are both NKLAM-dependent and NKLAM-independent mechanisms of tumor elimination *in vivo*. We previously demonstrated residual killing by NKLAM-deficient NK cells, suggesting that NKLAM is one of multiple factors contributing to the total amount of cytotoxicity mediated by NK cells [12]. Overall, these studies demonstrate that tumor cells that over-express UCKL-1 are more resistant to NK-mediated cytotoxicity *in vitro* and *in vivo*.

PCR analysis revealed that 3T3 cells have relatively low endogenous levels of UCKL-1. Due to their lower level of expression, we predicted that there would be a greater impact of UCKL-1 over-expression on cell growth and survival. UCKL-1 transfected 3T3 cells were significantly less susceptible to staurosporine-induced apoptosis than control transfected 3T3 cells. For quantitative proliferation studies, we generated a stable line of 3T3 cells expressing high levels of UCKL-1. These cells have a greater proliferative index than control-transfected cells. Therefore, over-expression of UCKL-1 enhances both proliferation and cell survival.

Other markers of proliferation are cyclin D and cyclin E. Both cyclins have been shown to be over-expressed in various cancers and are often associated with increased metastasis and decreased patient survival [28,29]. We found that UCKL-1 transfected K562 cells have higher levels of cyclin D and cyclin E than control-transfected cells. Cyclin D synthesis is initiated at G1 and cyclin E expression is highest at the G1-S transition, reaching an established threshold level before S phase [37–39]. The higher levels of cyclin D and cyclin E in UCKL-1 transfected K562 cells may be markers of enhanced proliferation. We were not able to quantitate increased proliferation in K562 cells transiently over-expressing UCKL-1 using either the cell proliferation dye efluor 670 or by tritiated thymidine incorporation. However, using 3T3 cells stably expressing UCKL-1, we found that these cells have a higher proliferative rate than control-transfected cells. As with K562, UCKL-1 transfected 3T3 cells have higher amounts of cyclin D and cyclin E than control-transfected cells. Increased expression of cyclin D and cyclin E in UCKL-1 transfected K562 and 3T3 cells is seen at both the protein and mRNA level. This suggests that higher levels of cyclins may contribute to a higher rate of proliferation in cells over-expressing UCKL-1.

High levels of cyclin D and cyclin E may also contribute to enhanced resistance to apoptosis in cells over-expressing UCKL-1. Studies have shown an association between cyclin E levels and resistance to apoptosis. Cyclin E is a marker of poor clinical outcome in breast cancer [40]. Cells over-expressing cyclin E are more resistant to trastuzumab, an anti-HER2/neu monoclonal antibody used to treat breast cancer [34]. The greater resistance to programmed cell death is associated with an increase in cyclin dependent kinase 2 activity. Cytoplasmic cyclin D1 has been shown to inhibit radiation-induced apoptosis and cyclin D1 over-expression causes resistance to some cytotoxic drugs [29,41,42]. UCKL-1 over-expression may trigger an increase in cyclin D and cyclin E expression, thereby protecting cells from apoptosis. This is consistent with our results illustrating a decrease in susceptibility of cells over-expressing UCKL-1 to drug and NK-mediated apoptosis. The higher levels of UCKL-1 observed in cancer cells may be a mechanism to enhance their survival by increasing their resistance to immune-mediated killing.

The involvement of UCKL-1 in proliferation, apoptosis, and susceptibility to NK-mediated cytolysis led us to examine NF-κB activity in tumor cells with varying levels of UCKL-1. NF-κB controls the transcription of a variety of genes associated with proliferation, apoptosis, immune functions and metastasis [20]. NF-κB activity affects NK cell number and activity as well as metastasis by inducing transcription of genes crucial for NK function and cytotoxicity [24,43]. By altering NF-κB activity, UCKL-1 expression may affect many elements of tumor progression. Both down-regulation and over-expression of UCKL-1 result in significant changes in NF-κB activity. UCKL-1 activates NF-κB in a dose-dependent manner. To address how UCKL-1 may be affecting NF-κB activity, we examined the translocation of NF-κB from the cytoplasm to the nucleus. In unstimulated cells, NF-κB is retained in the cytoplasm by inhibitors such as IκBα. Degradation of inhibitors allows the active form of NF-κB to translocate to the nucleus and bind DNA [44,45]. NF-κB is a protein complex with p50, p52, p65, c-Rel, and RelB subunits. The p65 subunit contains the transcriptional activation domain. We found more translocation of p65 to the nucleus in cells with higher UCKL-1 expression. IκBα, an inhibitory subunit in the NF-κB complex, plays a major role in the nuclear translocation of NF-κB. IκBα is phosphorylated, ubiquitinated, and then degraded, which releases NF-κB to translocate to the nucleus [20,21]. We found half the level of IκBα in cells over-expressing UCKL-1 than in control-transfected cells. This suggests that degradation of IκB is at least in part responsible for the increased translocation and greater functional activity of NF-κB in UCKL-1 transfected cells. Studies to further delineate the mechanism by which UCKL-1 affects the translocation and activity of NF-κB are in progress. Increased NF-κB activity in some tumors is associated with changes in expression of chemokines, cytokines, oncogenes, tumor suppressor genes, and apoptosis inhibitors, resulting in decreased apoptosis and increased growth. NF-κB also regulates the transcription of cyclin D and cyclin E [25,26,46,47].We found increased RNA levels of these cyclins in UCKL-1 transfected cells, consistent with transcriptional regulation. Differences in NF-κB localization are also observed in cancer cells. Breast cancer cells have increased nuclear localization of NF-κB and malignant melanoma cells have constitutive IκB kinase activation, resulting in rapid degradation of IκB and increased NF-κB translocation and activity [48–50]. The increased nuclear translocation and activity of NF-κB in cells over-expressing UCKL-1 may enhance tumor progression. This is consistent with both genomic and proteomic data demonstrating highest levels of UCKL-1 in the most aggressive, metastatic breast and prostate cancer cells [14,15]. Current studies are focused on further investigating the function of UCKL-1 in tumor growth and metastasis and determining the mechanism by which UCKL-1 influences NF-κB activity. Once the method of action of UCKL-1 is determined, it may be possible to design new cancer therapeutics that target UCKL-1 directly, or indirectly by enhancing NK and NKLAM function.
